# Employees’ pro-environmental behavior in an organization: a case study in the UAE

**DOI:** 10.1038/s41598-024-66047-4

**Published:** 2024-07-04

**Authors:** Nadin Alherimi, Zeki Marva, Khalid Hamarsheh, Ayman Alzaatreh

**Affiliations:** 1 Department of Industrial Engineering, American University of Sharjah, Sharjah, UAE; 2 Department of Mathematics and Statistics, American University of Sharjah, Sharjah, UAE

**Keywords:** Pro-environmental behavior, Green Entrepreneurial Orientation, Green Leadership, Environmental commitment, Green Human Resource Management, Structural equation modeling, Environmental economics, Statistics, Environmental impact

## Abstract

This study investigates the factors influencing employees’ pro-environmental behavior (PEB) within organizations in the United Arab Emirates (UAE), a nation with a strong policy focus on sustainability. Utilizing a questionnaire-based survey of 146 employees in an automotive division of a UAE company and structural equation modeling (SEM), the research examines the impact of green entrepreneurial orientation, green leadership, environmental commitment, and Green Human Resource Management (GHRM) on employees’ willingness to engage in eco-friendly practices at work. The findings reveal that GHRM and green leadership significantly influence employees’ green entrepreneurial orientation, which in turn, alongside environmental commitment, positively impacts PEB. These results emphasize the importance of integrating sustainability into organizational culture, leadership, and human resource practices to foster a workforce that actively participates in environmental initiatives, thereby contributing to the development of sustainable communities and enhancing stakeholder engagement. The study provides valuable insights into the specific factors that drive PEB in the UAE context, where national policies prioritize sustainability, highlighting that the importance of implementing green practices and promoting a supportive environment encourages employees and stakeholders to embrace environmental sustainability. The research also sheds light on the role of green entrepreneurial orientation, suggesting that empowering employees to develop innovative environmental solutions can be a key driver of PEB. The SEM analysis also confirmed the positive impact of GHRM and green leadership on green entrepreneurial orientation. Additionally, green entrepreneurial orientation and environmental commitment were found to significantly influence PEB. These results have practical implications for organizations in the UAE and beyond, emphasizing that by integrating eco-friendly practices and fostering stakeholder engagement, organizations can enhance their environmental performance, strengthen their reputation, and attract environmentally conscious customers and employees, contributing to the development of sustainable communities.

## Introduction

The increasing concern surrounding environmental issues has facilitated a paradigm shift in the business landscape, with sustainability becoming a major concern^1–3^. Environmental challenges, including climate change, ozone depletion, deforestation, ecosystem degradation, and biodiversity loss, have garnered widespread attention from the public and policymakers alike. In response to this heightened awareness, the imperative for businesses to adopt a more responsible approach towards environmental protection has become increasingly evident^4^. Organizations are now expected to integrate environmental considerations into their core business strategies, encompassing resource management, waste reduction, and the minimization of negative ecological impacts. This shift towards sustainability not only aligns with ethical imperatives but also serves as a strategic advantage, ensuring long-term viability and mitigating potential future risks^4^.

Within this context, employees' pro-environmental behavior (PEB) has emerged as a critical factor in achieving corporate sustainability goals^5^. PEB refers to the deliberate actions taken by employees to minimize their negative impact on the environment, both within and outside the workplace. These behaviors can range from simple actions like recycling and conserving energy to more complex initiatives like advocating for sustainable practices within the organization. Research has shown that employee’s PEB is not only beneficial for the environment but also positively impacts organizational performance, including financial outcomes, employee morale, and corporate reputation.

The existing body of literature indicates the importance of employees' pro-environmental behavior (PEB) in the workplace for enhancing an organization's environmental performance^5^. Despite previous research, various issues have not yet been thoroughly examined and properly understood. In this context, one of the studies proposes that an intriguing area of investigation is concerned with the impact of Green Human Resource Management (GHRM) on enhancing the pro-environmental performance of employees^6^. To promote the environmental performance of employees, companies must take into account the PEB of employees in conjunction with their GHRM practices. Organizations have succeeded in improving the PEB of employees by implementing GHRM practices^6^. Similarly, another work has concluded from the research in the hospitality sector that green HR practices assist employees in enhancing their eco-friendly behaviors^7^. Besides, a recent study reveals a positive relationship between GHRM practices and pro-environmental behavior in the Nigerian hospitality sector^8^. The results highlight the importance of green human capital as a mediating factor, suggesting that fostering employees' green skills and knowledge is key to promoting sustainable practices. Moreover, a study highlighted that by integrating GHRM practices that prioritize sustainability, manufacturing companies can effectively promote green behavior in the workplace, leading to improved environmental performance and contributing to a cleaner and more sustainable environment^9^. However, a study conducted in the industrial sector found that while GHRM predicts the PEB of employees, another crucial factor, namely employees’ “green self-efficacy,” still requires exploration^10^. As GHRM enhances employees’ skills and behavior, it can lead to the development of improved green entrepreneurship among employees. Furthermore, green entrepreneurship can serve as a predictor of employees’ PEB. This particular mechanism, which combines GHRM with green entrepreneurship, warrants further investigation, as it has the potential to bring about the most significant changes and improvements.

Another important factor influencing PEB is green leadership. Green leaders are those who prioritize environmental sustainability in their decision-making and actions^11^. They serve as role models for employees, demonstrating a commitment to environmental protection and inspiring others to follow suit. Green leadership can manifest in various ways, such as setting ambitious environmental goals, advocating for sustainable practices, and creating a supportive environment for employees to engage in PEB.

Environmental commitment, which refers to an individual's sense of responsibility and dedication to protecting the environment, is also a significant predictor of PEB^12^. Employees who are deeply committed to environmental causes are more likely to engage in PEBs, both at work and in their personal lives. This commitment can be fostered through various means, such as environmental education and awareness programs, opportunities for employees to participate in environmental initiatives, and recognition of their contributions to sustainability.

Furthermore, the literature on sustainable entrepreneurship within established firms, particularly those with significant environmental footprints, is limited^13^. Identifying the critical factors that enable pro-environmental and resilient entrepreneurship in these contexts is crucial for promoting green innovation and overcoming the challenges associated with implementing sustainable practices. Understanding the role of environmental commitment, a key factor in fostering PEB, is particularly relevant in this context.

Research has also established a link between corporate social responsibility (CSR), environmental commitment, and PEB, suggesting that employees' perceptions of their organization's commitment to sustainability influence their own pro-environmental actions^14^. This finding is consistent with social exchange theory^15^, which posits that employees reciprocate favorable organizational policies and practices with positive behaviors^16–20^. However, the specific mechanisms through which perceived CSR translates into PEB and the role of organizational culture in this process require further investigation.

To address these gaps in the literature, this study aims to develop and validate a comprehensive theoretical framework that examines the impact of green leadership, GHRM practices, green entrepreneurship, and environmental commitment on PEB. By investigating these factors in an integrated manner, this research seeks to contribute to a more holistic understanding of the determinants of PEB globally. The study will focus on a service-oriented organization in the UAE, a context where national policies actively prioritize sustainability, making it a particularly relevant setting for investigating PEB. The findings of this research will not only advance theoretical knowledge but also offer practical insights for organizations seeking to promote PEB and achieve their sustainability goals.

This study is motivated by the need to understand the complex interplay of factors that influence employees’ pro-environmental behavior in the unique context of the UAE, where sustainability is a national priority. The research aims to fill the gap in the literature by providing a comprehensive model of PEB that integrates green leadership, GHRM practices, green entrepreneurship, and environmental commitment. The target of this study is to offer practical recommendations for organizations in the UAE and beyond on how to effectively promote PEB among their employees, thereby contributing to a more sustainable future.

The remainder of this paper is structured as follows: section “Literature review” provides a comprehensive overview of the relevant literature and theoretical foundations, outlining the specific hypotheses to be tested. Section “Methodology” details the research methodology employed, including data collection and analysis procedures. Section “Discussion” presents a thorough discussion of the results, interpreting the theoretical and practical implications gained in this research. Finally, section “Summary, conclusion, and recommendations” concludes the study by summarizing the key findings, drawing conclusions, and offering recommendations for future research directions in this important and evolving field.

## Literature review

Environmental conservation and sustainability have emerged as prominent organizational goals in recent years, with companies actively seeking to align their operations with eco-friendly practices. Achieving environmental sustainability is contingent upon employees consistently engaging in pro-environmental behaviors (PEBs). These behaviors encompass a range of quantifiable actions that contribute to a greener workplace^21^, as well as employees' intentions to participate in sustainable activities^22^. Notably, PEBs are often considered voluntary, extra-role behaviors that employees undertake to benefit their organizations^23^. These behaviors can manifest in various ways, from resource conservation efforts like turning off lights and using double-sided printing, to waste management practices aimed at protecting the environment. As environmental concerns gain prominence, businesses are increasingly investing in employee programs to enhance environmental efficiency^24^. However, the success of such programs ultimately depends on the extent to which employees engage in PEBs^25^. The literature highlights the crucial link between PEB and organizational success, both financially and non-financially^21^. This research aims to delve deeper into this connection by examining the impact of various factors on PEB, including green entrepreneurship, green leadership, environmental commitment, and GHRM practices. By understanding these factors, organizations can gain valuable insights for fostering a culture of sustainability and maximizing the benefits of employee PEBs.

### Green Entrepreneurial Orientation

The literature suggests a link between employee pro-environmental behavior (PEB) and innovation within organizations. Environmental issues often require complex solutions, leading to a focus on environmental innovations and the factors influencing entrepreneurial intentions^26,27^. Green entrepreneurship, where employees engage in entrepreneurial activities that promote environmental sustainability, plays a crucial role in shaping their attitudes and behaviors, ultimately enhancing their pro-environmental conduct^28^.

The literature suggests a multifaceted relationship of green entrepreneurship, creativity, innovation, and PEB in employees. Green entrepreneurship, focused on identifying and addressing environmental issues through available opportunities, has been found to positively influence PEB^29^. This is likely due to the increased environmental awareness and problem-solving skills that employees develop through active engagement in green initiatives. Additionally, employee creativity and innovation have been identified as critical factors in the greening of organizations^23^. Employees who can generate novel ideas and effectively implement them to solve environmental problems contribute significantly to the development of sustainable practices. This suggests that creativity and innovation are not only valuable for organizational performance but also essential drivers of PEB. The empowering nature of green entrepreneurship further supports this connection. When employees with a green mindset are empowered to act on their ideas, they are more likely to initiate environmental projects and develop new ecological approaches, ultimately fostering PEB within the workplace^25^.

The literature highlights the significant influence of employee innovation capabilities on pro-environmental behavior (PEB). Studies indicate that employees leverage their knowledge to understand environmental concerns and develop solutions, such as pollution control programs and carbon emission reduction measures^30,31^. This impact extends beyond specific industries, as sectors like banking and universities can also benefit from employee knowledge sharing and empowerment to address environmental challenges. Furthermore, research emphasizes that PEB is not solely determined by traditional predictors but is also shaped by employee creativity, innovativeness, and tacit skills^25,32^. These factors enable employees to generate and implement novel ideas for environmental sustainability, contributing to a more comprehensive understanding of PEB. Additionally, green leadership skills have emerged as a critical factor influencing PEB in organizations. While the specific mechanisms through which green leadership affects PEB require further investigation, its importance in shaping organizational culture and promoting pro-environmental practices is evident.

### Green Leadership

The literature emphasizes the crucial role of leadership in shaping organizational behavior and outcomes. Leadership, defined as the ability to influence others towards achieving goals^33^, is often associated with individual traits like intellect and dominance^34^. Organizations that foster leadership development by recognizing and nurturing future leaders create environments conducive to employee initiative and innovation^35^. This is particularly relevant in the context of environmental sustainability, where supportive work environments and cultures have been shown to motivate employees to implement pro-environmental practices, such as recycling programs and continuous improvement initiatives^36^. Environmental leaders, who prioritize environmental considerations in decision-making and organizational processes, play a key role in promoting pro-environmental behavior (PEB) within organizations. While environmental leadership does not strictly adhere to any single theory, it often exhibits characteristics of transformational leadership, inspiring and motivating employees to embrace pro-environmental values and actions^37^.

The literature highlights a complex interplay between leadership, environmental leadership, and pro-environmental behavior (PEB) in organizations. Transformational leadership, sharing traits with environmental leadership, is often applied to environmental contexts due to its focus on both internal and external relationships and its influence on individual and organizational levels^38,39^. This is evident in behaviors such as creating a compelling environmental vision, raising awareness of environmental issues, and demonstrating personal commitment to environmental concerns. Environmental leaders, both at the individual and organizational levels, play a crucial role in promoting sustainable practices within organizations^40,41^. Individual leadership can emerge from any member, while organizational leadership involves implementing eco-friendly policies and cultivating a sustainable culture. Top management support and commitment are essential for the successful implementation of such practices. Leadership influences human resource management practices and contributes to environmental performance^42^. Different leadership styles, such as vision development, problem-solving, innovation, trust-building, conflict management, and resource utilization, can be employed to guide individuals toward achieving environmental goals^34,43^. Moreover, leadership outcomes, like fostering pro-environmental initiatives and encouraging employee engagement in environmental entrepreneurship, are linked to increased PEB^42^.

### Environmental Commitment

Individuals who establish a psychological connection with nature are more likely to demonstrate environmental commitment^44^. This phenomenon can be explained through interdependence theory and the commitment model. Interdependence theory highlights the factors influencing commitment between individuals, while the commitment model focuses on the development of commitment itself. Both theories suggest that individuals tend to exhibit commitment towards entities they rely on to fulfill their needs and desires. Thus, a strong psychological connection to nature, representing a form of reliance for emotional well-being and belonging, could foster environmental commitment. This commitment, in turn, is expected to translate into increased PEB as individuals act in accordance with their environmental values and sense of responsibility^45^.

The literature suggests a complex relationship between biospheric values, environmental commitment, and pro-environmental behavior (PEB). Biospheric value orientation, reflecting an individual's concern for the environment, has been found to predict environmental intentions, behaviors, and preferences^46^. Environmental commitment, the degree to which an individual is dedicated to environmental protection, is positively correlated with biospheric values^47^, indicating that individuals with strong environmental values are more likely to exhibit high levels of commitment. This commitment, in turn, has been shown to influence an individual’s willingness to act and make sacrifices for the environment^44,46,48^. Individuals with high environmental commitment are more likely to engage in pro-environmental behaviors and make choices that benefit the environment, such as consuming green products and adopting sustainable practices.

Based on the literature, a multifaceted relationship between environmental commitment, pro-environmental behavior (PEB), and several influencing factors. A study proposes that an individual's mental model of the environment can significantly impact their level of environmental commitment and subsequent PEB^49^. This implies that the way individuals perceive and conceptualize the environment influences their willingness to engage in pro-environmental actions. Furthermore, other factors like an individual's psychological connection to nature, adherence to interdependence and commitment theories, reliance on the environment, and biospheric values have been shown to predict environmental commitment and PEB^44–47^. A strong psychological connection to nature, for instance, can foster a sense of responsibility and belonging, increasing environmental commitment and promoting PEB.

### Green Human Resource Management

GHRM has emerged as a pivotal concept in the realm of sustainable business practices, encompassing human resource activities that explicitly address a firm’s environmental and ecological impact^50^. GHRM is intrinsically linked to an organization’s environmental policy and the ecological behaviors of its employees, highlighting the importance of aligning human capital with environmental objectives^51^. The significance of GHRM in the literature on sustainable human resource management lies in its comprehensive approach, which emphasizes the integration of environmental management practices into core business operations^6^. By acting as a pathway between human resource management and environmental management, GHRM reflects an organization’s strategic commitment to sustainability. This commitment necessitates top management’s active involvement in designing and implementing organizational processes and strategies that encourage employees to participate in environmentally conscious activities aimed at reducing emissions^52^. In essence, GHRM operationalizes an organization’s environmental management objectives through its human resource systems^53^. This includes incorporating environmental considerations into various HR functions such as performance management, incentives, training and development, recruitment and selection, and fostering a green entrepreneurship orientation among employees. Notably, research suggests that GHRM can enhance employees’ skills and behaviors, leading to the development of improved green entrepreneurship among employees^11^. Furthermore, green entrepreneurship can serve as a predictor of employees' PEB. This mechanism, which combines GHRM with green entrepreneurship, warrants further investigation as a potential catalyst for significant changes and improvements in PEB.

These hypotheses are grounded in the understanding that GHRM practices not only equip employees with the necessary knowledge and skills for environmentally responsible actions but also cultivate a sense of environmental awareness and commitment, ultimately leading to increased PEB both within and outside the workplace. Additionally, the mediating role of green entrepreneurship highlights the potential for GHRM to empower employees to become agents of change, further amplifying its positive impact on PEB.

By testing these hypotheses, this study aims to shed light on the complex relationship between green leadership, GHRM practices, green entrepreneurship, environmental commitment and PEB, providing valuable insights for organizations striving to achieve both environmental and economic sustainability. Based on the findings of the literature, the green aspects that are assumed to impact on PEB of employees are summarized in Table 1 along with the indicators of each green aspect. Additionally, Tables 2 and 3 illustrate a summary of the studies found in literature regarding the green aspects that influence the PEB of employees in organizations.

**Table 1:** Green aspects and indicators for measuring PEB of employees.

No	Factor	Indicating variables	Label
		Name	
1	Green Entrepreneurial Orientation	Importance of environmental preservation	GEO1
		Environmental Purchasing	GEO2
		Unsustainability reduction	GEO3
2	Green Leadership	Green project pro-activeness	GL1
		High-risk green project development	GL2
		Greenmarket leader	GL3
3	Environmental Commitment	Pride	EC1
		Pleasure	EC2
		Environmental values	EC3
4	Green Human Resource Management	Performance appraisal	GHRM1
		Promotions	GHRM2
		Rewards and compensations	GHRM3

**Table 2:**
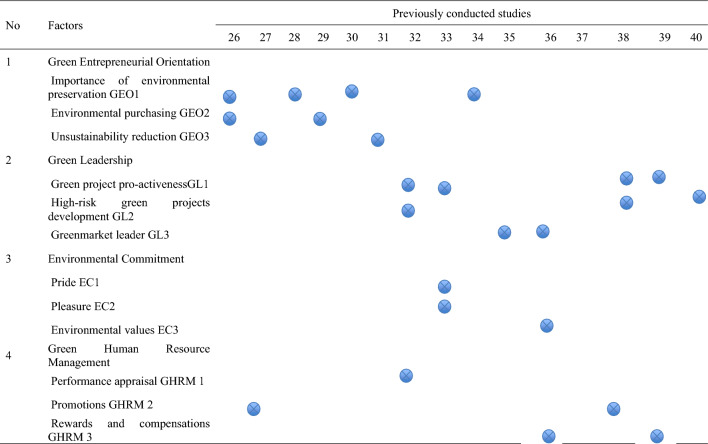
Green aspects for measuring PEB of employees.

**Table 3:**
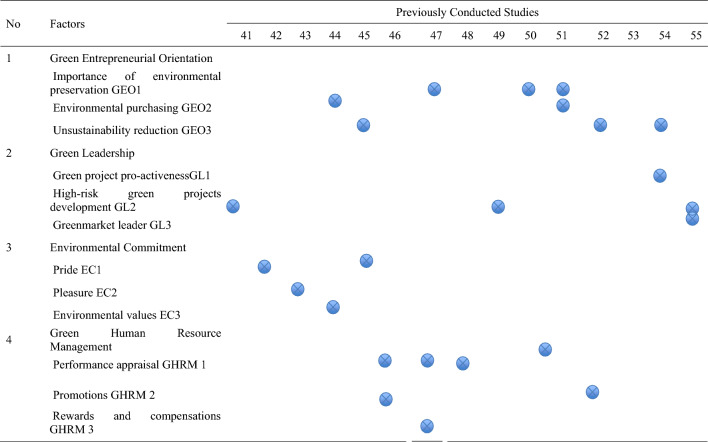
Green aspects for measuring PEB of employees.

## Methodology

This research adopted a multi-prolonged methodological approach which is initiated by identifying the problem, which is measuring the employees’ pro-environmental behavior (PEB) in a case organization in the UAE. Then, a comprehensive literature review to identify the green aspects related to PEB in existing research. Informed by the literature search, a questionnaire was developed and distributed to employees in a service-oriented organization in the UAE. The collected data underwent rigorous validation procedures to ensure the reliability and validity of the survey instrument. Kaiser–Meyer–Olkin (KMO) test measures the sampling adequacy of each variable in the model^54,55^. Cronbach’s alpha (α), measures of internal consistency, was employed due to its simplicity, interpretability, and widespread acceptance across various fields^56^. Its suitability for Likert-scale questionnaires further justified its selection over alternative measures. Additionally, convergent validity was established through composite reliability (CR) and average variance extracted (AVE) analyses, both specifically tailored for use in structural equation modeling (SEM)^57,58^. CR assessed the internal consistency of items measuring the same construct, while AVE determined the amount of variance in the construct explained by the items compared to measurement error.

**Figure 1:**
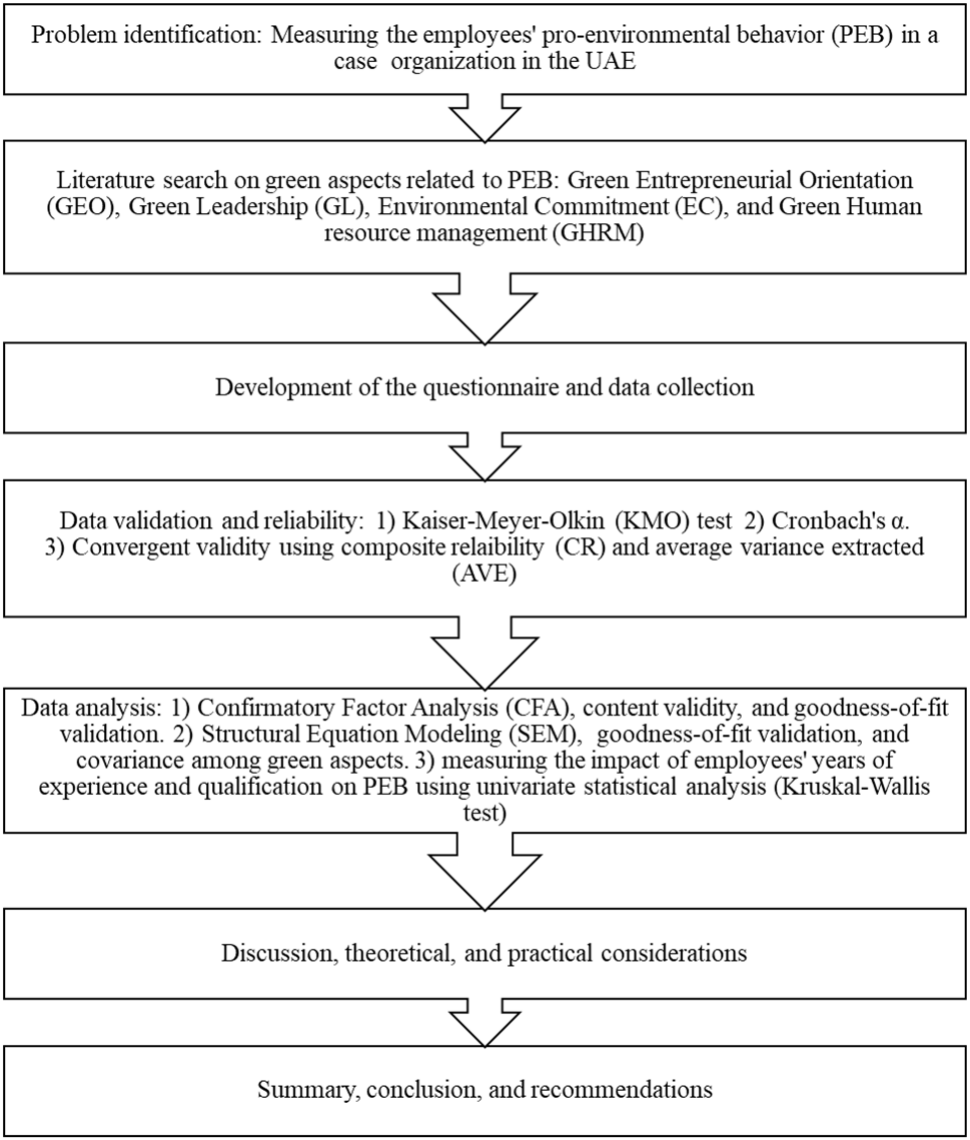
Research methodology.

After data validation, confirmatory factor analysis (CFA) was employed to identify and confirm the latent constructs underlying the green aspect influencing the PEB of employees in a case organization in the UAE, simultaneously testing the validity of the proposed measurement model^59^. CFA was chosen for its ability for model fit assessment and theory testing, which best suits this study as compared to item response theory and exploratory factor analysis. This is followed by content validity investigating standard estimates, to ensure the chosen indicators accurately represent the underlying constructs. Subsequently, structural equation modeling (SEM), by integrating factor analysis and path analysis, provided a robust framework for evaluating both the measurement model and the hypothesized structural relationships^60^. SEM was utilized to examine the impact of independent factors (Green Entrepreneurial orientation, Green Leadership, Environmental Commitment, and GHRM) on the dependent factor (PEB) followed by goodness-of-fit validation and measuring the impact of employees’ years of experience and qualification on PEB using univariate statistical analysis. Finally, the results and discussion, summary, conclusion, and recommendations were drawn (see Fig. 1).

As such, the research methodology was conducted in the following steps to ensure a systematic and reliable investigation.

Step 1: Problem identification—The main focus of the study is to shed light on measuring the PEB in a service-oriented organization in the UAE by designing a survey to collect responses and reviews from employees who participated in this research to evaluate the main effective factors that enhance and boost environmental awareness of the employees.

Step 2: Literature search—The literature review aided the study in developing an understanding of the most popular green aspects that impact on the successful PEB of employees in an organizational setting in the UAE.

Step 3: Questionnaire and data collection—An online questionnaire was conducted with employees and experts in an automotive division in the UAE to evaluate the factors identified in Table 1, as well as gain an in-depth understanding of their perceptions of the most essential factors affecting PEB in the UAE. The study followed snowball sampling approach^61^, which helps conduct research about people with specific traits to guide the study results, which include the opinions of employees and experts from an automotive division in the UAE.

Step 4: Data validation and reliability—The validity and reliability are crucial for any research conducting a questionnaire, if a questionnaire is not valid or reliable, the results will be flawed and cannot be used to make informed decisions or draw accurate conclusions. Therefore, researchers conducted sampling adequacy test through Kaiser–Meyer–Olkin (KMO) test to ensure the sample size is adequate. This is followed by the reliability measure through Cronbach’s α which measures how closely a set of indicators are related as a group (the relevant green aspect). Then, the convergent validity was measure through composite reliability (CR) which is a more consistent measure as compared to Cronbach’s alpha in measuring the internal consistency of the indicators on the green aspects, as well as the average variance extracted (AVE) which measures the amount of variance captured by the green aspects (independent factors) in relation to the amount of variance due to measurement error. This assists practitioners to ensure that the questionnaire is a trustworthy and accurate tool for measuring the construct it is intended to measure.

Step 5: Data analysis—This step involves a comprehensive assessment of the relationships between green aspects and PEB through confirmatory factor analysis (CFA) which was initially employed to verify a theoretical model of how specific indicators relate to broader green aspects, including Green Entrepreneurial orientation, Green Leadership, Environmental Commitment, and GHRM. The model's content validity was then examined by investigating standard estimates, ensuring the chosen indicators accurately represent the underlying constructs. Goodness-of-fit validation within the CFA statistically assessed how well the entire model aligns with observed data. Following this, structural equation modeling (SEM) was utilized to analyze causal relationships between independent factors (i.e., Green Entrepreneurial orientation, Green Leadership, Environmental Commitment, and GHRM) and the dependent factor, PEB. This step reveals the strength and significance of these relationships, providing insights into which green aspects most significantly impact PEB. Finally, another round of goodness-of-fit validation within the SEM context ensured the statistical soundness of the proposed relationships between green aspects and PEB. This rigorous approach not only validates the measurement model of green aspects but also tests their causal effects on PEB, offering both theoretical and practical implications for organizations aiming to foster a sustainable and attractive workplace. This is followed by univariate statistical analysis related to employees’ years of experience and qualification on PEB.

Step 6: Discussion—This section helped synthesizing the findings of the study in relation the impact of green aspects on PEB of employees in a service-oriented organization in the UAE, connecting them to existing knowledge, exploring their significance, and discussing the theoretical and practical implications of the study.

Step 7: Summary, conclusion, and recommendations—This section summarizes the key findings, draw conclusions based on the comprehensive methodology, and provides recommendations for further studies.

### Questionnaire design and data collection

According to the literature review in section “Literature review”, several studies discussed the employees’ PEB through several green aspects as shown in Tables 2 and 3. The green aspects include the green entrepreneurial orientation, green leadership, environmental commitment, and GHRM where each green aspect was assessed through three indicators. Each indicator was characterized by one question in the questionnaire by utilizing the five-point Likert scale for the green aspects were clearly explained to the employees and how they impact PEB. The sampling strategy followed a snowball sampling approach which helps conduct research about people with specific traits to guide the study results^62^. The questionnaire was distributed through google forms to all employees from different departments, professions, and experience levels in an automotive division of a group of companies as part of COP28 initiatives hosted by the UAE government. Thus, the company can evaluate, plan, and budget the required resources accordingly. As a result, 146 employees responded to the questionnaire.

### Respondents’ demographic information

The respondents’ (employees’) demographic information is shown in Table 4 below. It is shown that 146 employees participated in the questionnaire, of which, the male respondents accounted for 86.3% of the total employees as compared to the female employees with 13.7% of the total. Moreover, most of the respondents’ ages (46.58%) were between 25 and 30 years, which indicates that most of the respondents considered for measuring employees’ PEB are adult and mature employees. According to the employees’ qualification, most of the respondents completed their undergraduate degree with a 67.81% of the total, this implies a higher level of maturity in terms of PEB as the universities in the UAE continuously urge students to engage in green and environmentally friendly practices. Moreover, the years of experience of most of the employees’ range between 1 to 7 (30.14%), 8 to 15 (32.19%), 16 to 23 (30.82%), and 24 and above (6.85%); this shows that most of the respondents were those with 8–15 years of experience in their profession and will be aware of the suitable environmental behaviors to be considered in their field of expertise. For the employment level, most of the respondents were from the intermediate level (45.89%) who are mostly supervisors and can promote other employees to adopt PEB at work.

**Table 4:** Respondents’ demographic information.

Demographic information	Frequency	Percentage (%)
Gender		
Male	126	86.30
Female	20	13.70
Employee age group		
18–24	15	10.27
25–30	68	46.58
31–40	34	23.29
41 or older	29	19.86
Employee qualification		
High school	9	6.16
Diploma	17	11.64
College	99	67.81
Masters	21	14.38
Employee years of experience		
1–7	44	30.14
8–15	47	32.19
16–23	45	30.82
24 and above	10	6.85
Employment level		
Entry level	19	13.01
Intermediate	67	45.89
Mid-level	50	34.25
Senior or executive level	10	6.85

### Data analysis

This section analyzes the questionnaire data through the following steps: (1) data validation and reliability measures; (2) confirmatory factor analysis; (3) structural equation modeling; (4) univariate statistical analysis. A detailed data analysis is discussed below.

#### Data validation and reliability

This step is a crucial initial step to be assessed prior to CFA and SEM analysis, as it tests the collected data’s validity and reliability. Table 5 summarized the validity and reliability measures which consist of the standardized correlation coefficient with the total observed indicators within the same green aspect, KMO test, Cronbach’s α, and CR, and AVE. Furthermore, the sample size adequacy for SEM was assessed using the KMO test. A KMO value within the range of 0.7–1 signifies sufficient sample size^62^. In this study, the KMO value was 0.839, confirming the adequacy of the sample for SEM analysis. Moreover, Cronbach’s α is a measure of the internal consistency, which measures how closely related a set of indicators are as a group (the relevant green aspect)^56^. A Cronbach’s α value of 0.7 or higher is considered acceptable^63^, and according to Table 5, the Cronbach’s α values for all green aspects are between 0.83 and 0.91, which is acceptable. Similarly, the CR measures the internal consistency of the indicators on the green aspects, however, it is more consistent than Cronbach’s α^57^. A CR value of 0.7 or above is considered acceptable^64^, and according to Table 5, the CR values are between 0.84 and 0.91 which is acceptable. Furthermore, the AVE measures the amount of variance captured by the green aspects (independent factors) in relation to the amount of variance due to measurement error^58^. An AVE value of 0.5 or above is considered acceptable^65^, and according to Table 5, the AVE values are between 0.64 and 0.77 which is acceptable. Consequently, the questionnaire results are valid and reliable.

**Table 5:** Data validation, reliability, and CFA estimates.

Green aspect	Correlation with total	Cronbach's α	CR	AVE	CFA standardized parameter estimates	CFA parameter p-value
Green Entrepreneurial Orientation		0.8503	0.8512	0.6560		
Importance of preservation (GEO1)	0.715386				0.8138	< 0.0001
Environmental purchasing (GEO2)	0.722901				0.8097	< 0.0001
Unsustainability reduction (GEO3)	0.725055				0.8062	< 0.0001
Green Leadership		0.8884	0.8905	0.7310		
Project pro-activeness (GL1)	0.733247				0.7898	< 0.0001
High-risk projects (GL2)	0.813784				0.8911	< 0.0001
Market leader (GL3)	0.802399				0.8805	< 0.0001
Environmental Commitment		0.8297	0.8398	0.6371		
Pride (EC1)	0.647168				0.7212	< 0.0001
Pleasure (EC2)	0.739666				0.8584	< 0.0001
Environmental values (EC3)	0.698038				0.8090	< 0.0001
Green Human Resource Management		0.9084	0.9087	0.7684		
Performance appraisal (GHRM1)	0.820940				0.8869	< 0.0001
Promotions (GHRM2)	0.798758				0.8499	< 0.0001
Rewards and compensations (GHRM3)	0.830465				0.8924	< 0.0001

#### Confirmatory factor analysis

This subsection analyzes the CFA results (Table 5), conducts content validity and tests the CFA model’s goodness of fit for validation. The CFA’s standardized parameter estimates (loadings) are considered as a measure or the content validity which should have a value of 0.7 or higher, and according to Table 5, the estimates are between 0.72 and 0.89 which suggests that all the indicators are significant, and the content validity is confirmed in related to the green aspects. For validation purposes, the p-value for each indicator is calculated in Table 5, which shows that all indicators have a p-value < 0.05 which confirms that all indicators are significant to the green aspects.

The goodness-of-fit tests for the CFA model include: standardized root mean square error (SRMSR) which measures the mean absolute value of the covariance residuals, which should be 0.05 or less, goodness of fit index (GFI) which measures the fit between the hypothesized model and the observed covariance matrix and should be 0.9 or above, the adjusted goodness of fit index (AGFI) corrects the GFI, which is affected by the number of indicators of each latent variable which should be 0.9 or above, and Bentler comparative fit index (CFI) takes into account the sample size and avoids the underestimation of the fit which should be 0.9 or above. Table 6 shows that SRMSR = 0.0578 which is acceptable, GFI = 0.9209, AGFI = 0.8715, and CFI = 0.9725. Hence, the goodness of fit values reveals an acceptable fit. These findings demonstrate both the strength of the individual indicators and the overall validity of the CFA model, paving the way for a more in-depth analysis using SEM.

**Table 6:** CFA goodness of fit criteria.

Criteria	Value
Standardized RMR (SRMR)	0.0578
Goodness of Fit Index (GFI)	0.9209
Adjusted GFI (AGFI)	0.8715
Bentler Comparative Fit Index (CFI)	0.9725

#### Structural equation modeling (SEM)

**Figure 2:**
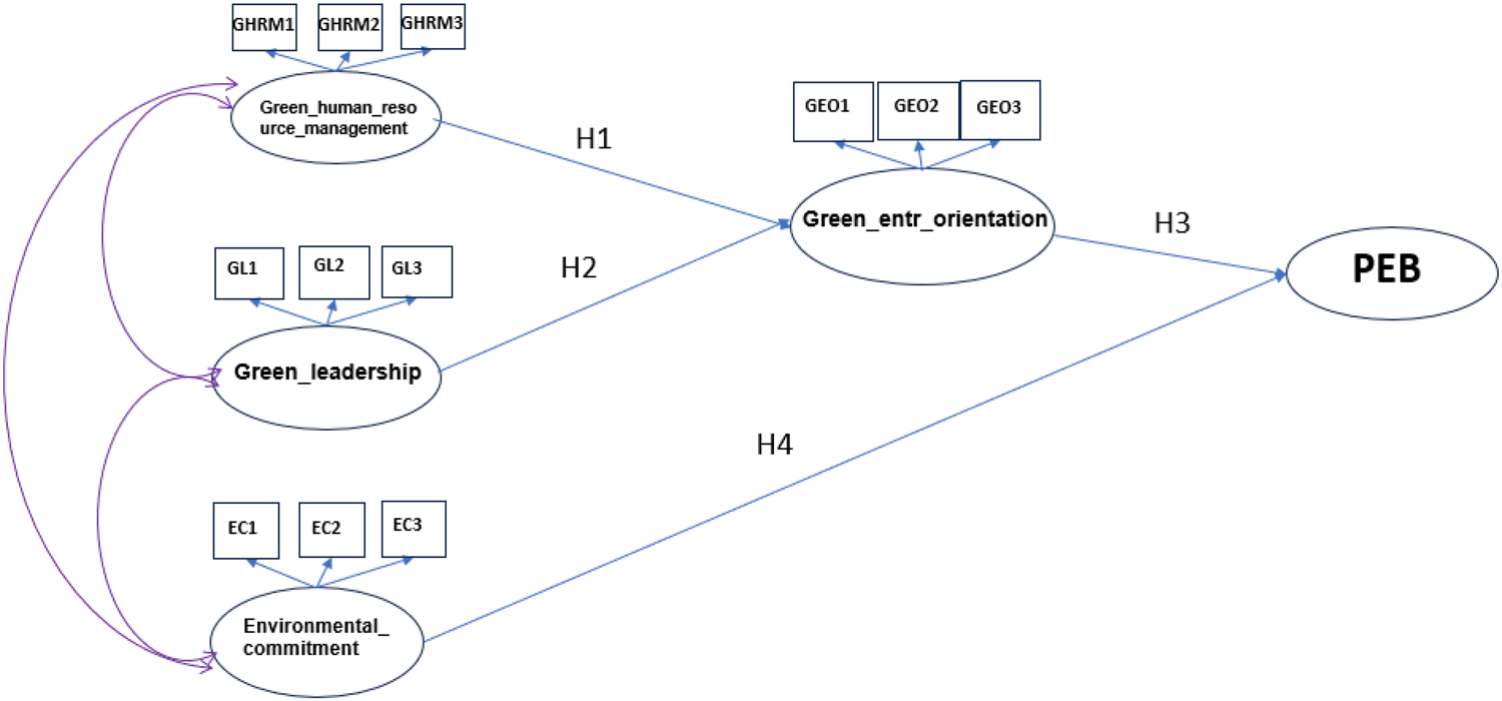
SEM model for PEB.

In this subsection, and according to the literature review, it was assumed that “GHRM” and “green leadership” have a positive impact on “green entrepreneurial orientation” of employees, and “green entrepreneurial orientation” and “environmental commitment” have a positive impact on PEB of employees as shown in SEM model in Fig. 2. The model aims to measure and test the PEB of 146 employees in an automotive division of a group of companies as part of COP28 initiatives hosted by the UAE government. According to Fig. 2, the following hypotheses are assumed:

**H1**: Green human resources management has a positive impact on green entrepreneurial orientation of employees in an organization.

**H2**: Green leadership has a positive impact on green entrepreneurial orientation of employees in an organization.

**H3**: Green entrepreneurial orientation has a positive impact on employees’ PEB in an organization.

**H4**: Environmental Commitment has a positive impact on employees’ PEB in an organization.

**H5**: There is a significant correlation among green human resources management, green leadership, and environmental commitment.

The SEM MODEL in Fig. 2 was fitted to the data using PROC CALIS in SAS software. The results of the fit in terms of green aspects (independent factors) and PEB (dependent factor) are depicted in Table 7. Based on the path analysis in Table 7, all the indicators are significant in measuring each green aspect with a p-value < 0.05. Furthermore, the goodness of fit indices depicted in Table 8 show that the SRMR = 0.0670 which is acceptable, GFI = 0.9035, AGFI = 0.8485, and CFI = 0.9590 which are all close to 0.9. Hence, the goodness of fit values reveals an acceptable fit. Furthermore, to check the positive impact of each green aspect on employees’ PEB, H_1_–H_4_ were tested. Consequently, the “green human resource management” and “green leadership” have a positive impact on “green entrepreneurial orientation” of employees with p-values of < 0.0001 and 0.0037, respectively; and “green entrepreneurial orientation” and “environmental commitment” have a positive impact on PEB of employees with p-values of 0.0229 and 0.0002, respectively. Moreover, the correlations among the exogenous factors (GHRM, green leadership, and environmental commitment) are tested (H_5_) as shown in Table 9. The results in Table 9 reveal that there is significant correlation between “green human resource management” and “green leadership” and between “green leadership” and “environmental commitment” with p-values of < 0.0001 and 0.0021. However, there is no significant correlation between “green human resource management” and “environmental commitment” with a p-value of 0.4254.

**Table 7:** SEM path list.

Path			Standardized estimate	P-value for the unstandardized estimate
Green_entr_orientation	===>	GEO1	0.8333	< 0.0001
Green_entr_orientation	===>	GEO2	0.7895	< 0.0001
Green_entr_orientation	===>	GEO3	0.8012	< 0.0001
Green_leadership	===>	GL1	0.7946	< 0.0001
Green_leadership	===>	GL2	0.8866	< 0.0001
Green_leadership	===>	GL3	0.8799	< 0.0001
Environmental_commitment	===>	EC1	0.7162	< 0.0001
Environmental_commitment	===>	EC2	0.8775	< 0.0001
Environmental_commitment	=== >	EC3	0.7925	< 0.0001
Green_human_resource_management	===>	GHRM1	0.8860	< 0.0001
Green_human_resource_management	===>	GHRM2	0.8510	< 0.0001
Green_human_resource_management	===>	GHRM3	0.8936	< 0.0001
Green_human_resource_management	===>	Green_entr_orientation	0.4425	< 0.0001
Green_leadership	===>	Green_entr_orientation	0.2924	0.0037
Environmental_commitment	===>	PEB	0.1927	0.0229
Green_entr_orientation	===>	PEB	0.3073	0.0002

**Table 8:** SEM goodness of fit criteria.

Criteria	Value
Standardized RMR (SRMR)	0.0670
Goodness of Fit Index (GFI)	0.9035
Adjusted GFI (AGFI)	0.8485
Bentler Comparative Fit Index (CFI)	0.9590

**Table 9:** SEM covariance matrix among green aspects.

	Green human resource management	Green leadership	Environmental commitment
Green human resource management		0.58791 < 0.0001	− 0.07438 0.4254
Green leadership			0.27159 0.0021

#### Univariate Statistical analysis

For further analysis, this subsection performs a Kruskal–Wallis (K-W) test which is a non-parametric statistical test that measures the differences among three or more independent sample groups on a single non-normally distributed variable (PEB) by comparing medians^66^. The K-W test was performed to investigate the impact of the employees’ years of experience (1–7, 8–15, 16–23, and 24 or above) and employees’ qualification (high school, diploma, college, and masters) on PEB. The K-W test for the differences between employees in terms of years of experience suggests that there is no evidence of difference in terms of the median PEB of employees, with a p-value of 0.2852. Moreover, the K-W test was performed to investigate the impact of the employees’ qualification on PEB. The K-W test for the differences between employees in terms of qualification suggests that there is no evidence of difference in terms of median PEB of employees, with a p-value of 0.5783.

### Institutional review board statement

As per American University of Sharjah guidelines, an Institutional Review Board (IRB) form was submitted to the Office of the Institutional Research, and an official approval was obtained to collect the necessary data.

## Discussion

In this paper, the green aspects that are impacting on the employees’ PEB were investigated to study the relationship between some crucial green factors and Employee’s PEB. These dimensions are green entrepreneurial orientation of the organization, green leadership of the organization, environmental commitment of employees and GHRM of the organization. This study developed and tested a model that focuses on understanding the impact of the above green aspects (independent factors) on PEB (dependent factor), which results in a comprehensive conclusion on the significant factors that impact on PEB of employees in an organization in the UAE. The general findings of the study supported the proposed framework.

SEM approach was utilized to evaluate each green aspect’s impact on PEB of employees in a case organization in the UAE. It was found that “Green Human Resource Management” has a positive impact on “Green Entrepreneurial Orientation” of employees. Hence, H_1_ is accepted. This finding was in line with the findings of a previous study^67^, which concluded that “green human resource management” improves the “green entrepreneurial orientation” of employees directly by incorporating environmental considerations in the recruitment and selection process. Research findings indicate that organizations integrating GHRM practices witness a significant increase in employees’ green entrepreneurial abilities, with a reported improvement in innovative environmental ideas generated by staff. Moreover, the study highlights that companies implementing GHRM strategies observe a rise in employee motivation towards PEB, leading to a notable enhancement in overall environmental performance within the organization. Additionally, the research emphasizes that GHRM initiatives contribute to an increase in employees’ green self-efficacy, empowering them to tackle environmental challenges creatively. These statistics demonstrate the tangible impact of GHRM on fostering green entrepreneurial orientation among employees, ultimately driving sustainable practices and outcomes in organizational settings.

Similarly, the proposed SEM model reveals that “green leadership” has a positive impact on “green entrepreneurial orientation” which reveals that H_2_ is accepted. This result builds on a previous work^68^, which discloses that the role of “green leadership” on “green entrepreneurial orientation” is important. Green leadership plays a pivotal role in shaping the direction and success of green entrepreneurial orientation within Indian organizations. By embodying environmentally conscious values and practices, green leaders inspire and motivate employees to embrace sustainability initiatives, drive innovation, and foster a culture of environmental responsibility. Green leaders set the tone for the organization by championing eco-friendly practices, setting ambitious sustainability goals, and demonstrating a commitment to reducing environmental impact. Their visionary approach to sustainability not only influences day-to-day operations but also guides strategic decision-making towards green entrepreneurship. Through effective communication and role modeling, green leaders create a shared sense of purpose and direction, aligning employees with the organization’s environmental goals and fostering a collective commitment to sustainability. Moreover, green leadership encourages a mindset of continuous improvement and adaptation to changing environmental challenges, driving organizational agility and resilience in the face of sustainability issues. Overall, the positive impact of green leadership on green entrepreneurial orientation lies in its ability to cultivate a culture of innovation, environmental stewardship, and sustainable growth, positioning organizations as leaders in the green economy and driving long-term success in a rapidly evolving environmental landscape. The emphasis on green leadership aligns with broader societal trends towards sustainability communities and responsible business practices, making it a critical factor for organizations seeking to succeed in the twenty-first century.

It is also worth mentioning that the SEM model showed that “green entrepreneurial orientation” has a positive impact on PEB. This result is also supported by a previous work^67^, which concluded that “green entrepreneurial orientation” enhances the PEB which is in line with the results of this study (i.e., H_3_ is accepted). Green entrepreneurial orientation plays a pivotal role in driving PEB among employees within organizations. By fostering a culture of innovation, creativity, and environmental consciousness, green entrepreneurial initiatives empower individuals to actively engage in sustainable practices. Employees with a green entrepreneurial mindset are more likely to identify environmental challenges as opportunities for positive change, leading to the development of innovative solutions and eco-friendly practices, which also fosters other stakeholders’ engagement. This orientation encourages employees to think beyond traditional approaches and explore novel ways to reduce environmental impact, such as implementing recycling programs, adopting energy-efficient technologies, or promoting sustainable resource management. Furthermore, green entrepreneurial orientation instills a sense of ownership and responsibility towards environmental stewardship, motivating individuals to proactively participate in green initiatives and advocate for eco-friendly policies within the organization. By nurturing a workforce that embraces green entrepreneurship, organizations can not only enhance their environmental performance but also cultivate a collective commitment to sustainability that extends beyond the workplace. This synergy between green entrepreneurial orientation and PEB not only drives positive environmental outcomes but also fosters a culture of environmental responsibility and innovation that is essential for addressing contemporary environmental challenges.

Lastly, the study concluded that “environmental commitment” has a positive impact on PEB of employees. Thus, H_4_ is accepted. Consequently, previous research done^14,69^ discovered evidence in favor of a favorable relationship between the perceived dedication to the environment and PEB. Environmental commitment refers to an individual’s dedication and responsibility towards the environment. It is a crucial factor in fostering PEB, which consciously seeks to minimize the negative impact of one’s actions on the natural and built world^70^. A study found that higher levels of commitment to the environment and greater inclusion of nature in the self separately predicted higher levels of PEB, even when controlling for social desirability and ecological worldview. This suggests that individuals who are more committed to the environment and feel a greater connection with nature are more likely to engage in behaviors that benefit the environment^71^. Another research focused on action research developing positive interactions between humans and the environment. More precisely, it reviewed commitment-making strategies and the effects of binding communication on the adoption of PEBs such as waste sorting, recycling, non-activist behaviors in the public sphere and energy saving. The study found that commitment, disagreement and binding communication can strengthen the positive characteristics of interactions between humans and the environment and, thereby, improve quality of life^72^. Building on that, it was found that higher levels of commitment to the environment and greater inclusion of nature in the self separately predicted higher levels of PEB. Besides, it was shown in a study that of rural residents, 91% are concerned about deforestation, 92% about plastic pollution and 90% about air pollution, with rural residents also being more likely to engage in personal behaviors to reduce their impacts on the climate^73^. This suggests that individuals who are more committed to the environment and feel a greater connection with nature are more likely to engage in behaviors that benefit the environment either in daily life activities or at workplace. This indicates a strong correlation between environmental commitment and PEB. By fostering a strong commitment to the environment, this encourages more sustainable communities and contributes to environmental preservation leading to stakeholders’ engagement to green practices^74^. However, it’s important to note that environmental commitment works best when combined with other factors such as environmental consciousness, social norms, and effective communication strategies^75,76^.

Overall, this study provides constructive insights into the factors that impact the PEB employees in workplace. This study emphasizes the impact of green entrepreneurial orientation, green leadership, environmental commitment, and GHRM on employees' PEB which fosters the willingness of employees and all stakeholders to engage in eco-friendly practices at work. These results highlight the importance of integrating sustainability into organizational practices to foster sustainable communities in the workforce which are committed to environmental responsibility. These represent solutions that address the needs of today's businesses while also safeguarding the environment and gaining widespread support^77^. Furthermore, the findings highlight that when employees perceive their organization as environmentally responsible, they are more likely to engage in PEB, creating positive feedback that reinforces the organization's commitment to sustainability and strengthens its relationships with stakeholders.

In the UAE context, where sustainability is a national priority, the study's findings are particularly relevant. Organizations that prioritize sustainability and actively engage with stakeholders can align themselves with the nation's vision for a sustainable future^78^. This alignment contributes to the overall well-being of the community, as well as positions the organization as a responsible corporate citizen, enhancing its reputation and fostering positive relationships with government agencies and regulatory bodies. By integrating sustainability into their operations and engaging with stakeholders, organizations in the UAE can play a crucial role in building sustainable communities and contributing to the nation's environmental goals.

### Theoretical implications

This research on employees' PEB within UAE organizations significantly advances the theoretical understanding of the complex relationship between workplace green aspects and employees' perceptions of their own professional and personal growth. By highlighting the pivotal roles of green entrepreneurial orientation, green leadership, environmental commitment, and GHRM, this study contributes robustly to the evolving concept of sustainability within the organizational context. The findings illuminate a critical shift in employee awareness shedding light that organizations integrating eco-innovation and sustainable practices not only benefit the environment but also enhance employees' perceived career advancement opportunities^79,80^. This aligns with global trends wherein employees increasingly seek value-aligned employers^81^, underscoring the increasing importance of sustainability in the modern workplace. This is particularly noticeable in the UAE, where national policies actively prioritize sustainability, further amplifying the significance of these findings for both academic and practitioner audiences.

### Practical implications

The practical implications of this study for organizations are diverse. Promoting green practices such as GHRM and Green Leadership is crucial, as these practices not only contribute to environmental sustainability but also positively impact employees' Green Entrepreneurial practices, fostering a culture of innovation and proactive environmental stewardship. Moreover, promoting environmental commitment among employees through awareness programs, training, and a sustainability-focused work culture can significantly enhance PEB, leading to a more engaged and environmentally conscious workforce. By encouraging innovative and proactive green initiatives, organizations can harness the positive impact of Green Entrepreneurial Orientation on PEB, driving the development and implementation of eco-friendly solutions.

The insights from this study can guide policymaking in organizations, enabling the design of policies that incentivize green practices and foster environmental commitment among employees. Organizations that successfully implement these practices can contribute to building sustainable communities through their environmentally responsible actions, enhancing their reputation and stakeholders’ engagement^82,83^. This can lead to a competitive advantage by attracting environmentally conscious customers and employees, fostering a positive brand image, and strengthening relationships with stakeholders who value sustainability. While implementing these practices requires commitment and strategic planning, the potential benefits for the organization, its stakeholders, and the environment are substantial, making it a worthwhile investment for a sustainable future.

## Summary, conclusion, and recommendations

This research investigates the factors influencing employees' PEB within organizations in the UAE. It examines the impact of green entrepreneurial orientation, green leadership, environmental commitment, and GHRM on employees' PEB which fosters their willingness to engage in eco-friendly practices at work. The study employed a questionnaire-based survey of 146 employees in an automotive division of a UAE company, utilizing structural equation modeling (SEM) to analyze the data. The study’s findings reveal that GHRM and green leadership significantly influence employees’ green entrepreneurial orientation. In turn, both green entrepreneurial orientation and environmental commitment positively impact employees' PEB. These results underscore the importance of integrating sustainability into organizational culture, leadership, and human resource practices to foster a workforce that actively participates in environmental initiatives. Consequently, this research provides valuable insights into the specific factors that drive PEB in the UAE context, where national policies prioritize sustainability. It highlights the importance of not only implementing green practices but also cultivating a supportive environment that encourages employees to embrace and champion environmental sustainability. The study also sheds light on the role of green entrepreneurial orientation, suggesting that empowering employees to develop innovative environmental solutions can be a key driver of PEB. The SEM analysis confirmed the positive impact of GHRM (p < 0.0001) and green leadership (p = 0.0037) on green entrepreneurial orientation. Additionally, green entrepreneurial orientation (p = 0.0229) and environmental commitment (p = 0.0002) were found to significantly influence PEB. These results have practical implications for organizations in the UAE and beyond.

The study emphasizes that integrating eco-friendly practices benefits both the environment and employee development. It highlights the importance of green leadership, environmental commitment, GHRM practices, and green entrepreneurial initiatives. To achieve this, the study provides several recommendations for organizations, particularly in the UAE. Firstly, organizations should prioritize comprehensive GHRM strategies. This entails integrating environmental considerations throughout all HR functions, from recruitment to performance management. Secondly, fostering a culture of environmental commitment is crucial. This involves open communication about the importance of sustainability, recognizing employees' PEBs, and providing opportunities for participation in green initiatives, this drives sustainable communities. Thirdly, promoting green leadership at all levels is essential. Green leaders act as champions for sustainability, inspiring employees and driving green stakeholder engagement. Finally, organizations should encourage and support green entrepreneurship. This means providing resources and platforms for employees to develop and implement innovative environmental solutions. By adopting these recommendations, organizations can create a work environment that fosters not only environmental well-being but also enhanced stakeholder engagement, satisfaction, and overall well-being. This ultimately positions them as environmentally responsible businesses with a competitive edge in attracting and retaining top talent.

Moreover, future research in this area could explore additional dimensions of green aspects that may influence employees' awareness and adaptability to PEB measures. For instance, investigating the impact of work atmosphere, direct managers/supervisors, financial situations, and social factors on employees' PEB could provide further insights into the complex interplay between organizational dynamics and environmental behaviors. Additionally, extending the study to different business divisions and comparing results across various sectors could offer a more comprehensive understanding of the factors influencing PEB in different organizational contexts. Furthermore, validating the findings in other countries and assessing the potential impact of geographic location on employees' awareness of PEB could help broaden the generalizability of the results and provide valuable cross-cultural insights into the relationship between green aspects and employees' environmental behaviors. By expanding the scope of research in this field and exploring new dimensions of green aspects, future studies can contribute to the ongoing discourse on sustainability in the workplace and offer practical recommendations for organizations seeking to enhance their environmental initiatives and promote a culture of environmental responsibility among employees.

## Data Availability

The data presented in this study are available on request from the corresponding author.
