# Author Correction: Employees’ pro-environmental behavior in an organization: a case study in the UAE

**DOI:** 10.1038/s41598-024-80625-6

**Published:** 2024-12-12

**Authors:** Nadin Alherimi, Zeki Marva, Khalid Hamarsheh, Ayman Alzaatreh

**Affiliations:** 1 Department of Industrial Engineering, American University of Sharjah, Sharjah, UAE; 2 Department of Mathematics and Statistics, American University of Sharjah, Sharjah, UAE

Correction to: *Scientific Reports* 10.1038/s41598-024-66047-4, published online 04 July 2024

The original version of this Article contained an error in the name of Ayman Alzaatreh, which was incorrectly given as Ayman Alzaaterh.

The original Article has been corrected.

